# Dynamics of Virus-Specific Memory B Cells and Plasmablasts following Viral Infection of the Central Nervous System

**DOI:** 10.1128/JVI.00875-18

**Published:** 2019-01-04

**Authors:** Jeffrey R. Atkinson, Mihyun Hwang, Angel Reyes-Rodriguez, Cornelia C. Bergmann

**Affiliations:** a Department of Neurosciences, Lerner Research Institute, Cleveland Clinic Foundation, Cleveland, Ohio, USA; b School of Biomedical Sciences, Kent State University, Kent, Ohio, USA; Loyola University Medical Center

**Keywords:** B cell responses, central nervous system infections, coronavirus, germinal centers, neuroimmunology, viral encephalomyelitis

## Abstract

Humoral responses within the central nervous system (CNS) are common to many neurotropic viral infections, with antibody (Ab)-secreting cells (ASC) contributing to local protection. However, a role for virus-specific memory B cells (Bmem) within the CNS is poorly explored due to lack of robust phenotypic or functional identification in mice. This study takes advantage of the progeny of mice expressing tamoxifen-inducible Cre recombinase (Cre-ERT2) under the *Aicda* promoter crossed with Rosa26-loxP-tdTomato reporter mice (AID^Cre^-Rosa26^tdTomato^) to monitor B cells having undergone activation-induced cytidine deaminase (AID)-mediated somatic hypermutation (SHM) following neurotropic coronavirus infection. AID detection via tdTomato expression allowed tracking of virus-specific ASC and Bmem in priming and effector sites throughout infection. In draining lymph nodes, tdTomato-positive (tdTomato^+^) ASC were most prevalent prior to germinal center (GC) formation, but total tdTomato^+^ B cells only peaked with robust GC formation at day 14 p.i. Moreover, their proportion of Bmem dominated over the proportion of ASC throughout infection. In the CNS, tdTomato^+^ cells started emerging at day 14 p.i. While they initially comprised mainly Bmem, the proportions of ASC and Bmem became similar as tdTomato^+^ B cells increased throughout viral persistence. Delayed tamoxifen treatment demonstrated ongoing CNS recruitment of tdTomato^+^ B cells, mainly ASC, primed late during GC reactions. Overall, the data support the idea that virus-induced B cells exhibiting SHM require peripheral GC formation to emerge in the CNS. Ongoing GC reactions and regional signals further regulate dynamics within the CNS, with preferential maintenance of tdTomato^+^ B cells in spinal cords relative to that in brains during viral persistence.

**IMPORTANCE** The prevalence and role of antigen-specific Bmem in the CNS during viral encephalomyelitis is largely undefined. A lack of reliable markers identifying murine Bmem has made it difficult to assess their contribution to local antiviral protection via antigen presentation or conversion to ASC. Using reporter mice infected with neurotropic coronavirus to track virus-specific Bmem and ASC, this report demonstrates that both subsets only emerge in the CNS following peripheral GC formation and subsequently prevail. While early GC reactions supported preferential Bmem accumulation in the CNS, late GC reactions favored ASC accumulation, although Bmem outnumbered ASC in draining lymph nodes throughout infection. Importantly, virus-specific B cells undergoing sustained GC selection were continually recruited to the persistently infected CNS. Elucidating the factors governing temporal events within GCs, as well as regional CNS cues during viral persistence, will aid intervention to modulate CNS humoral responses in the context of infection and associated autoimmune pathologies.

## Abstract (precis)

The prevalence and role of antigen-specific Bmem in the CNS during viral encephalomyelitis is largely undefined. A lack of reliable markers identifying murine Bmem has made it difficult to assess their contribution to local antiviral protection via antigen presentation or conversion to ASC. Using reporter mice infected with neurotropic coronavirus to track virus-specific Bmem and ASC, this report demonstrates that both subsets only emerge in the CNS following peripheral GC formation and subsequently prevail. While early GC reactions supported preferential Bmem accumulation in the CNS, late GC reactions favored ASC accumulation, although Bmem outnumbered ASC in draining lymph nodes throughout infection. Importantly, virus-specific B cells undergoing sustained GC selection were continually recruited to the persistently infected CNS. Elucidating the factors governing temporal events within GCs, as well as regional CNS cues during viral persistence, will aid intervention to modulate CNS humoral responses in the context of infection and associated autoimmune pathologies.

## INTRODUCTION

Neurotropic viral infections are associated with the accumulation of multiple B cell subsets within the central nervous system (CNS) whose composition alters with time, indicating ongoing turnover and differentiation (1–3). During infection with a gliatropic mouse hepatitis virus (MHV) derived from the JHM strain (JHMv2.2-1), IgD-positive (IgD^+^) (naive/transitional) B cells prevail early, but they decrease coincident with increasing proportions of CD138^+^ antibody (Ab)-secreting cells (ASC) and CD138^−^ IgG^+^ isotype-switched memory B cells (Bmem) as germinal centers (GCs) are formed in draining lymph nodes (1, 2). Whether the turnover in the CNS involves replacement by newly recruited B cells derived from the periphery or local expansion/differentiation of B cells recruited during acute infection is largely unresolved. While ASC in the blood are transiently migratory following antigen (Ag)-driven differentiation, Bmem recirculate for extended periods of time (4, 5). This supports circulating Bmem as a potential replenishing source of ASC in the CNS during persistent infections. However, their contribution to humoral immunity in the CNS following infection or autoimmunity remains poorly characterized.

Ag-specific B cells develop into ASC through two distinct pathways. In the first, Ag-activated B cells rapidly differentiate into short-lived ASC in extrafollicular foci (6–9). In the second pathway, B cells activated by Ag and receiving CD4 T cell help at the T cell-B cell follicle border form follicular GCs, where they undergo affinity maturation and isotype switching, ultimately generating Ag-specific Bmem, as well as long-lived IgG ASC. The generation of these IgG ASC and Bmem is significantly impaired in the absence of GCs (10–14). GC reactions are marked by class-switched Ig and somatic hypermutation (SHM), thereby imprinting long-lived ASC and Bmem (15–18). These processes are both mediated by the enzyme activation-induced cytidine deaminase (AID), which introduces mutations in DNA by deaminating the cytidine base to create uracil. Uracil-DNA glycosylase and apurinic endonuclease then act to excise uracil, and the gaps are filled in by DNA repair mechanisms (12, 19, 20). Based on its SHM-inducing activity, AID expression constitutes a B cell-specific marker of Ag-induced affinity maturation. While the vast majority of AID-dependent affinity maturation occurs within GCs, resulting in class-switching to IgG (21), there is evidence for extrafollicular, GC-independent SHM resulting in the production of Ag-specific ASC and Abs, including those of the IgM isotype (12, 22).

Following gliatropic MHV CNS infection, T and B cell responses are initiated in cervical lymph nodes (CLN), and local CNS Ab production is crucial for preventing recrudescence of persisting virus following initial T cell-mediated immune control (23, 24). Using the gliatropic MHV-JHMv2.2-1 variant, we have recently demonstrated that B cells in the CNS transition from a predominantly naive/early-activated IgD^+^ IgM^+^ population at day 7 p.i. to an IgD-negative (IgD^−^) IgM^+^ and increasingly IgG^+^ population, including CD138^+^ ASC, by day 21 p.i. (1, 2). Moreover, the B cell coreceptor CD19, which lowers the Ag-specific activation threshold and promotes peripheral GC formation, is required for accumulation of CD138^+^ ASC in the CNS (14). These data suggested that the vast majority of plasmablasts and IgD^−^ B cell subsets in the CNS have undergone SHM driven by viral Ag-specific B cell receptor (BCR) activation and do not comprise bystander B cells recruited via Ag-independent proinflammatory signals. However, identification of virus-specific ASC or Bmem generally depends on enzyme-linked immunosorbent spot assay (ELISPOT) techniques using virus or viral lysates as the capture Ag, potentially underestimating their numbers. Measurement of virus-specific Bmem is especially biased by culture conditions, as B cells require nonspecific stimulation *in vitro* to convert into ASC for subsequent quantitation by ELISPOT (25, 26).

To better characterize the proportions of virus-specific Bmem and ASC accumulating in the CLN and the CNS following viral encephalomyelitis, we took advantage of mice expressing tamoxifen-inducible Cre recombinase (Cre-ERT2) under the *Aicda* promoter crossed with Rosa26-loxP-tdTomato reporter mice to obtain progeny in which AID-expressing cells can be identified by fluorescence following tamoxifen administration (4, 27). Analysis of humoral responses to protein Ag in AID^Cre^-Rosa26^EYFP^ mice confirmed that the vast majority of enhanced yellow fluorescent protein (EYFP)-expressing B cells were indeed specific for the immunizing Ag (4). These dually transgenic reporter mice are thus suitable tools to phenotypically monitor the dynamics and tissue distribution of B cells having undergone virus-induced, AID-mediated SHM. This study used the MHV-A59 strain, a neurotropic MHV that is less pathogenic than JHMv2.2-1, to determine the frequency, longevity, and distribution of virus-specific ASC and Bmem in the CLN and CNS of infected AID^Cre^-Rosa26^tdTomato^ mice using the CD19^+^ tdTomato^+^ IgD^−^ CD138^+^ and the CD19^+^ tdTomato^+^ IgD^−^ CD138^−^ phenotype, respectfully. Tamoxifen administration at the onset of infection and throughout day 28 p.i. revealed that tdTomato^+^ B cells only accumulated in the CNS following peripheral GC formation and continued well into the chronic infection phase. Early GC-independent tdTomato^+^ ASC in the CLN did not appear to migrate to the CNS. Notably, an overall larger proportion of tdTomato^+^ B cells accumulated earlier and at higher frequencies in spinal cords than in brains. While Bmem dominated the tdTomato^+^ population in CLN throughout GC activity, they vastly exceeded ASC at early but not later stages of viral persistence. The administration of tamoxifen during chronic disease, starting at day 20 p.i., revealed that ∼50% of ASC and ∼25% of Bmem were recruited from later peripheral GC reactions by 28 days p.i., accounting for nearly the entire increase in virus-specific B cells observed in the CNS between days 21 and 28 p.i. Overall, the results show that the vast majority of ASC recruited to both the brain and spinal cord were virus specific, with limited accumulation of ASC with heterologous specificity. In contrast, the fraction of virus-specific cells within the Bmem population was substantially higher in spinal cords than in the brain. These data indicate that B cell subset accumulation during the persistent phase of infection is controlled by peripheral GC-driven events, as well as CNS regional signals.

## RESULTS

### Virus-specific tdTomato^+^ ASC preceded GC B cells and GC formation.

Following infection with gliatropic MHV-JHMv2.2-1, adaptive immune responses are initiated within CLN, consistent with lymphatic CNS drainage into this site (28). Moreover, virus-specific ASC measured by ELISPOT peak at ∼day 14 p.i., coincident with defined anatomical GC formation in CLN (1, 29). In contrast, the peak in total ASC monitored phenotypically precedes virus-specific ASC by ∼7 days p.i. (23). Importantly, both total and virus-specific ASC only start emerging in the CNS at day 14 p.i. and increase thereafter. We therefore questioned whether early ASC expansion is driven by Ag-independent innate immune signaling, as previously shown for influenza virus and West Nile virus (WNV) infection (30, 31), or whether it is Ag driven but insufficient to monitor via ELISPOT assay. AID^Cre^-Rosa26^tdTomato^ mice were thus treated with tamoxifen coincident with MHV-A59 inoculation to jointly track the emergence of tdTomato^+^ cells as a marker for Ag-primed Bmem (CD19^+^ tdTomato^+^ IgD^−^ CD138^−^) and ASC (CD19^+^ tdTomato^+^ IgD^−^ CD138^+^) in CLN. Naive mice treated with tamoxifen 2 days prior to analysis were used as controls to assess baseline tdTomato^+^ B cells.

**FIG 1:**
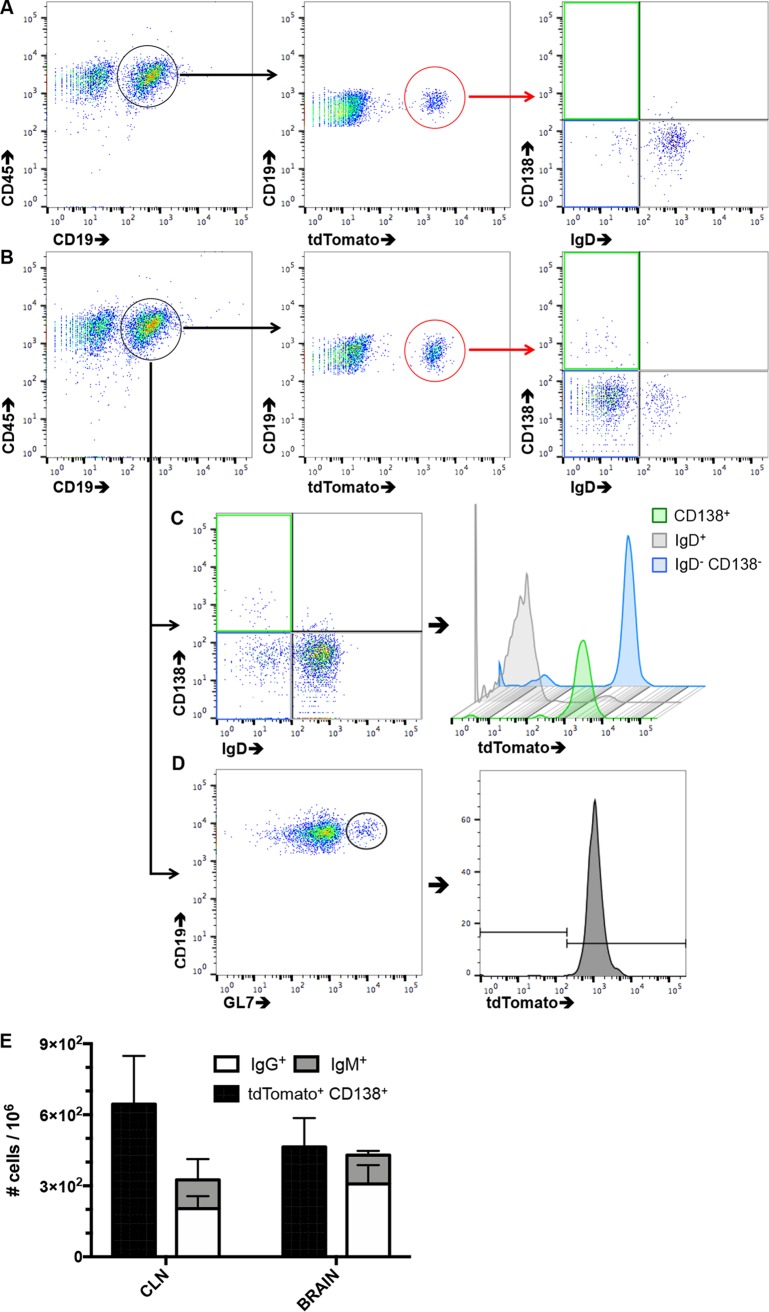
Gating strategy for quantification of AID-induced, tdTomato^+^, virus-specific B cell subsets in CLN via flow cytometry. Naive mice were treated with tamoxifen 2 days prior to analysis and MHV-infected mice from day 0 throughout day 14 prior to analysis. (A, B) Representative flow cytometry plots showing gating on CD19^+^ B cells from naive mice (A) or infected mice at 14 days p.i. (B). Following gating on tdTomato^+^ cells, the relative frequencies of B cell subsets were determined via expression of CD138 and IgD. (C) Subsets within CD19^+^ cells were distinguished and evaluated for the frequency of tdTomato^+^ cells. (D) GC B cells (GL7^+^) were identified within the IgD^−^ CD138^−^ subset and evaluated for tdTomato^+^ cells. Data are from individual mice representative of 4 to 8 individual mice. (E) Frequencies of tdTomato^+^ CD138^+^ ASC determined by flow cytometry compared to IgG^+^ and IgM^+^ ASC determined by ELISPOT at day 14 p.i. in the CLN and brain. Data represent the mean value ± SEM for 3 individual mice from a single experiment.

The flow cytometry gating strategy depicting tdTomato^+^ cells within total B cells and B cell subsets is shown in Fig. 1. tdTomato^+^ B cells in CLN of naive mice treated with tamoxifen constituted ∼1.5% of total CD19^+^ B cells (Fig. 1). Of these, only ∼35% were IgD^−^, indicative of basal GC reactivity. The minor proportion of basal tdTomato^+^ cells is consistent with a small fraction of GL7^+^ cells, driven by constitutive activation by endogenous Ag (4, 32). Untreated mice showed no AID-driven tdTomato reactivity.

Following infection, the frequency of tdTomato^+^ B cells within total CD19^+^ B cells increased to ∼5% at day 14 p.i., with the vast majority being IgD^−^ and only a small population expressing the ASC marker CD138 (Fig. 1A). Evaluation of tdTomato expression within total IgD^−^ CD138^−^ or CD138^+^ cells indicated the vast majority were recently induced to express AID (Fig. 1C). This was confirmed by monitoring the expansion of B cells expressing GL7, an activation marker for pre-GC and GC B cells (33). The fraction of GL7^+^ cells was only slightly smaller than that of tdTomato^+^ cells at day 14 p.i. Moreover, virtually all GL7^+^ cells were tdTomato^+^ (Fig. 1D), consistent with GC formation (4, 32). To confirm that the majority of tdTomato^+^ CD138^+^ ASC were indeed virus specific, we directly compared the frequencies of CD138^+^ tdTomato^+^ B cells obtained by flow cytometry to virus-specific ELISPOT analyses in CLN and brains at day 14 p.i. (Fig. 1E), when virus-specific ASC peak in CLN and emerge within the CNS following MHV-JHMv2.2-1 infection (4, 14, 32). In this experiment, the sum of virus-specific IgG- and IgM-secreting ASC approximated the number of tdTomato^+^ CD138^+^ cells at both anatomical sites, supporting their virus specificity.

**FIG 2:**
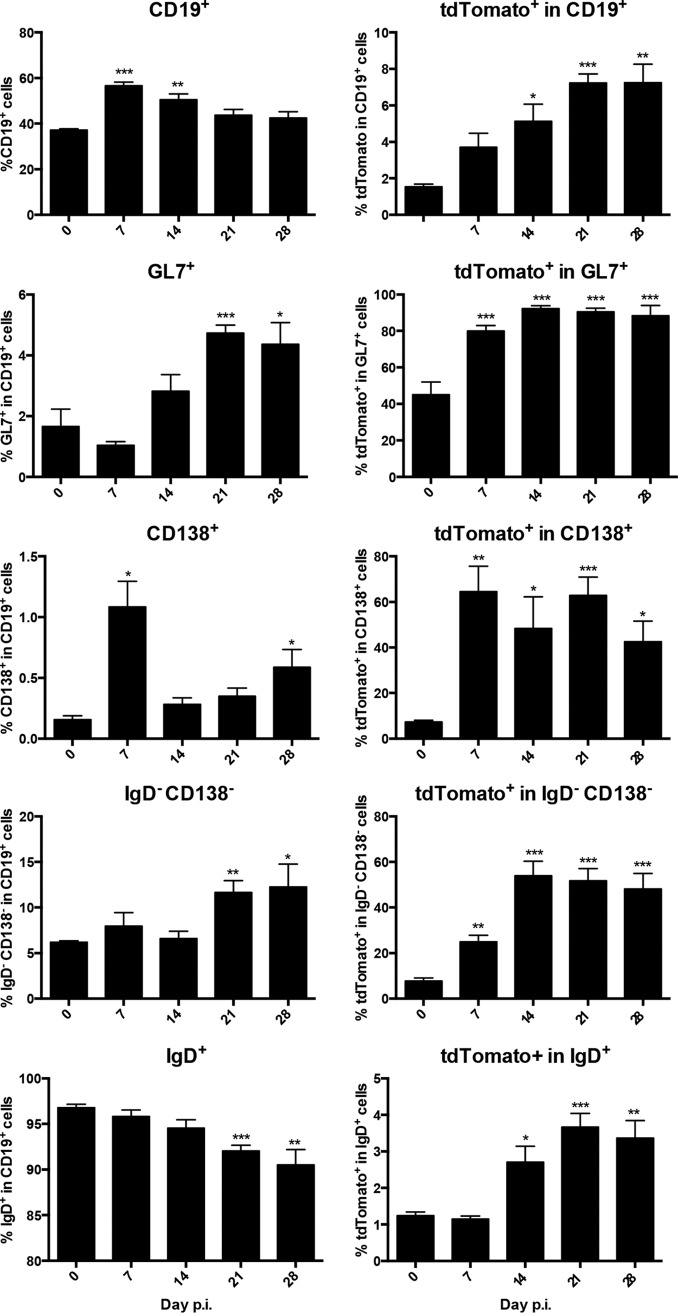
Frequencies of total and virus-specific tdTomato^+^ B cell subsets in CLN throughout MHV infection. B cell phenotypes in CLN were monitored by flow cytometry. Left, frequencies of CD19^+^ B cells within total CLN cells or of B cell subsets within the CD19^+^ B cell population at indicated times p.i. Right, frequencies of tdTomato^+^ cells within the indicated B cell populations. “0” indicates naive mice. Data represent the mean values ± SEM for individual mice from 2 separate experiments, each comprising 3 to 4 individual mice per time point. Statistically significant differences from the results for naive mice (day 0 p.i.), determined by unpaired *t* test, are denoted as follows: *, *P* < 0.05; **, *P* < 0.01; ***, *P* < 0.001.

**FIG 3:**
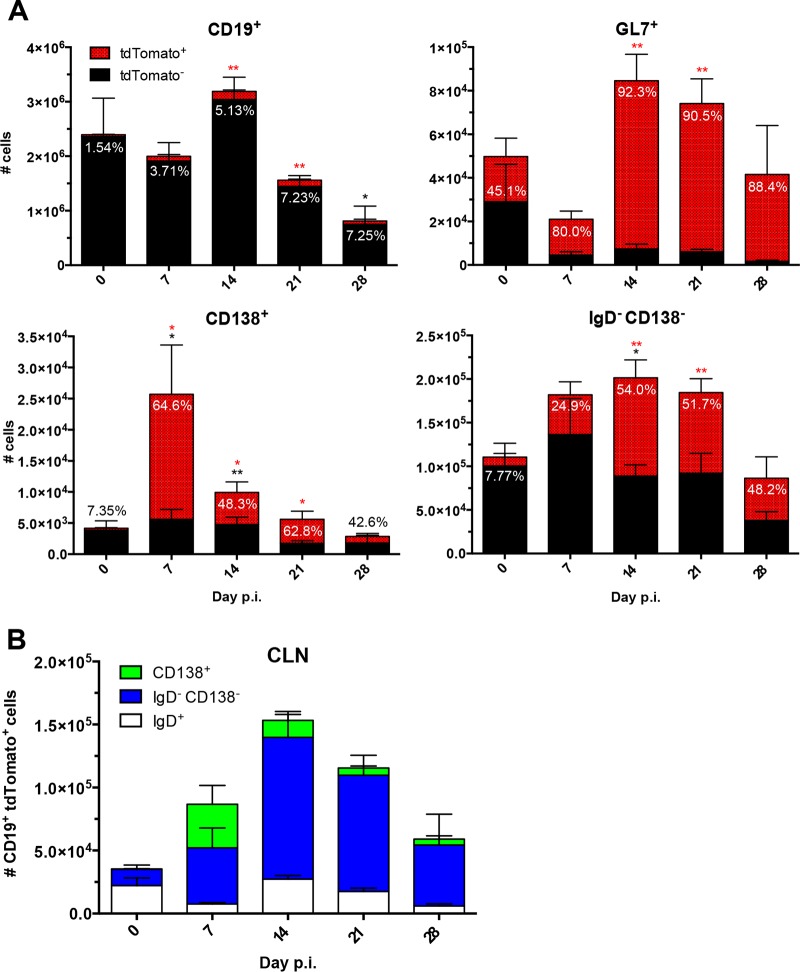
Peak numbers of virus-specific tdTomato^+^ B cells coincide with GC formation in the CLN. (A) Numbers of CD19^+^ B cells, CD138^+^ ASC, GL7^+^ GC phenotype B cells, and IgD^−^ CD138^−^ B cells in CLN following MHV infection. Data for tdTomato-negative (tdTomato^−^) cells are represented in black, and data for tdTomato^+^ cells are shown in red; the relative percentage of tdTomato^+^ cells within each defined B cell population is indicated above or within the bar. (B) Numbers of CD19^+^ tdTomato^+^ CLN cells subdivided into the relative numbers of CD138^+^, IgD^−^ CD138^−^, and IgD^+^ B cell subsets. Data are from the same mice used in the experiment whose results are shown in Fig. 1 and represent the mean values ± SEM for individual mice from 2 separate experiments, each comprising 3 to 4 individual mice per time point. Statistically significant differences from the results for naive mice (day 0 p.i.), determined by unpaired *t* test, are denoted as follows, with asterisks in red or black denoting significant differences in the tdTomato^+^ or total population, respectively: *, *P* < 0.05; **, *P* < 0.01; ***, *P* < 0.001.

Having confirmed expansion of tdTomato^+^ B cells with a largely IgD^−^ phenotype following virus CNS infection, we monitored tdTomato^+^ B cell subsets in CLN throughout acute infection (day 7 p.i.) into the persistent phase (days 14 to 28 p.i.). The proportion of tdTomato^+^ cells within total B cells was already increased at day 7 p.i. compared to that in naive mice, steadily rose to ∼7% by day 21 p.i., and remained stable thereafter (Fig. 2). The GL7^+^ B cell fraction was not increased until day 14 p.i. and reached maximum levels of ∼5% at day 21 p.i. (Fig. 2). Moreover, the fraction of GL7^+^ B cells expressing tdTomato increased to 80% by day 7 p.i. and plateaued at ∼90% by day 14 p.i. (Fig. 2). These data are consistent with the formation of well-defined GC at day 14 p.i. and segregation into structurally mature GC defined by dark- and light-zone domains by day 21 p.i. following infection with the JHMv2.2-1 virus variant (1). Moreover, the results confirmed AID-dependent SHM and class switch recombination (CSR), typical of GC B cells, and showed maintenance of GC activity out to day 28 p.i. during chronic infection. The increased proportion of GL7^+^ cells by day 14 p.i., as well as their tdTomato expression, support the idea that induction of GL7^+^ is specifically induced by viral Ag (Fig. 2 and 3). Contrasting with the progressively increasing proportions of tdTomato^+^ and GL7^+^ cells after day 14 p.i., CD138^+^ ASC proportions within CLN B cells peaked at day 7 p.i. (Fig. 2), suggesting that they were largely GC independent. Moreover, the fraction of ASC rapidly declined and only slowly increased again by day 28 p.i. (Fig. 2), similar to previous results during MHV-JHMv2.2-1 infection (14, 23). Surprisingly, however, 50% to 60% of ASC at day 7 p.i. were tdTomato^+^, compared to ∼5% in naive mice, and these percentages were sustained throughout day 28 p.i. (Fig. 2). These results are consistent with virus-activated AID expression and SHM prior to robust GC formation. However, it could not be discerned whether tdTomato^+^ ASC at later times comprised a distinct or more differentiated GC-derived subset. Importantly, the kinetics of IgD^−^ CD138^−^ Bmem did not mirror those observed for ASC. Total Bmem did not show increased frequencies within CD19^+^ CLN B cells in infected compared to naive mice until day 21 p.i. (Fig. 2). Bmem and ASC exhibited similar fractions of tdTomato^+^ cells in naive CLN (∼7%). However, unlike ASC, Bmem did not demonstrate a rapid increase in the frequency of tdTomato^+^ cells at day 7 p.i. Despite this initial divergence, the tdTomato^+^ fraction in Bmem increased to ∼50% at day 14 p.i. and remained stable out to day 28 p.i. (Fig. 2). The production of Ag-primed ASC and Bmem thus displayed discrete kinetics in CLN following MHV infection. Finally, while the fraction of tdTomato^+^ cells in phenotypically naive IgD^+^ cells increased by day 14 p.i. and remained elevated compared to that in naive mice, it remained <4%, suggesting rapid differentiation into IgD^−^ memory cells (Fig. 2) (1).

Figure 3 provides an overview of the temporal kinetics of absolute numbers of tdTomato^+^ cells relative to those of GL7^+^ GC B cells, CD138^+^ ASC, and IgD^−^ CD138^−^ Bmem throughout infection. Total CD19^+^ B cells were stable throughout day 14 p.i. and decreased thereafter, reflecting overall decreased cellularity of CLN following intracranial (i.c.) MHV infection. Although the percentages of tdTomato^+^ B cells slowly increased throughout days 7 to 21 p.i. (Fig. 2), the absolute numbers of tdTomato^+^ cells were most abundant at day 14 p.i., reflecting peak total CD19^+^ cells (Fig. 3A). The numbers of GL7^+^ B cells, with the vast majority being tdTomato^+^, mirrored the kinetics of GC formation (1). Total ASC peaked at day 7 p.i. (∼2.0 × 10^4^), prior to maximal GL7^+^ B cells (∼8.0 × 10^4^), reflecting peak CD138^+^ percentages within the B cell population. By day 28 p.i., ASC declined rapidly, reaching only ∼2 × 10^3^ (Fig. 3A). The high proportion (∼65%) of ASC expressing tdTomato at day 7 p.i. thus surpassed AID-expressing ASC numbers during maximal GC differentiation after day 14 p.i. (Fig. 3A). These early ASC are consistent with a population of largely GC-independent, IgM^+^, long-lived ASC, which have been described to exhibit AID-dependent affinity maturation in other models (12).

In stark contrast to ASC, Bmem demonstrated a progressive increase in tdTomato^+^ cell numbers in the CLN, peaking at day 14 p.i. (∼1.1 × 10^5^) and incrementally decreasing by day 28 p.i. (∼4.8 × 10^4^) (Fig. 3A). However, the frequency of tdTomato^+^ cells within Bmem remained constant during chronic infection. The decrease in tdTomato^+^ Bmem supports the notion of egress into circulation following peak GC formation. Interestingly, the dynamics of tdTomato^+^ Bmem in the CLN closely resemble those of GL7^+^ GC B cells, suggesting a relatively constant production of Ag-primed Bmem throughout the formation and contraction of GCs (Fig. 3A). However, as was the case with ASC, it remains unclear whether the tdTomato^+^ Bmem are more differentiated or exhibit increased affinity. The numbers of tdTomato^+^ IgD^+^ B cells were consistently low (<3.0 × 10^4^) (data not shown).

The distribution of B cell subsets within CLN tdTomato^+^ B cells is summarized in Fig. 3B. The total numbers of CD19^+^ tdTomato^+^ B cells increased significantly by day 14 p.i., slowly declined until day 21, and reached background levels by day 28 p.i. Of note, at day 14 p.i., they comprised mainly IgD^−^ CD138^−^ Bmem (∼72%) and a smaller proportion of CD138^+^ ASC (∼8%). The large proportion of Bmem relative to that of ASC was maintained throughout day 28 p.i. Despite overall lower levels of tdTomato^+^ ASC relative to the levels of Bmem, ASC kinetics following GC formation mirrored those of virus-specific IgG ASC determined by ELISPOT following heterologous MHV-JHMv2.2-1 infection (14).

### tdTomato^+^ cells localized predominantly to GCs in CLN.

**FIG 4:**
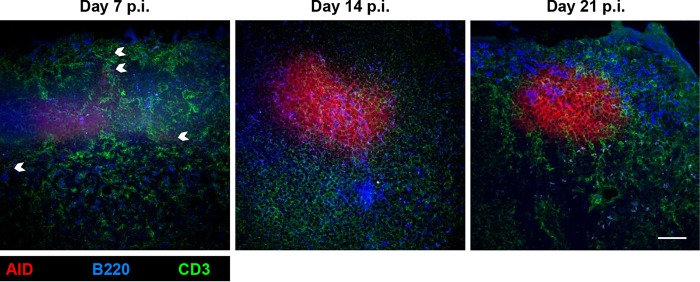
tdTomato^+^ cells in CLN form robust GCs by day 14 p.i. Representative fluorescent immunohistochemical images of CLN sections stained for B cells (B220, blue), T cells (CD3, green), and endogenous tdTomato expression (red), taken at ×40 magnification from projected Z-stacks of 6.69 μm in depth. White arrows indicate extrafollicular tdTomato^+^ B cells. Figures are representative of the results for 2 mice per time point. Scale bar = 50 μm.

To affirm that the majority of tdTomato^+^ B cells were indeed imprinted by GC reactions, CLN sections from infected, tamoxifen-treated AID^Cre^-Rosa26^tdTomato^ mice were assessed for anatomical GC formation at days 7, 14, and 21 p.i., corresponding to times from emergence of GCs to mature GC formation following CNS infection with the more pathogenic gliatropic MHV-JHM strain (1). At day 7 p.i., AID^Cre^-Rosa26^tdTomato^ mice already exhibited small foci of tdTomato^+^ B cells within follicles, indicative of early GC formation. tdTomato^+^ B cells were also evident at extrafollicular locations, consistent with GC-independent expansion of tdTomato^+^ ASC (Fig. 4). By day 14 p.i., tdTomato^+^ B cells formed large foci typical of GC morphology within follicles; these structures were maintained through day 21 p.i. (Fig. 4) and day 28 p.i. (data not shown). Overall, these kinetics of GC formation are similar to those of infection with MHV-JHM2.2v-1 (1). Moreover, although tdTomato^+^ ASC and Bmem (Fig. 3B) had already emerged at day 7 p.i., the maximum GL7^+^ tdTomato^+^ populations nevertheless coincided with mature GC formation (Fig. 3), suggesting that the vast majority of virus-specific B cells were GC derived.

### Virus-specific ASC and Bmem accumulated progressively in the CNS following peripheral GC formation.

**FIG 5:**
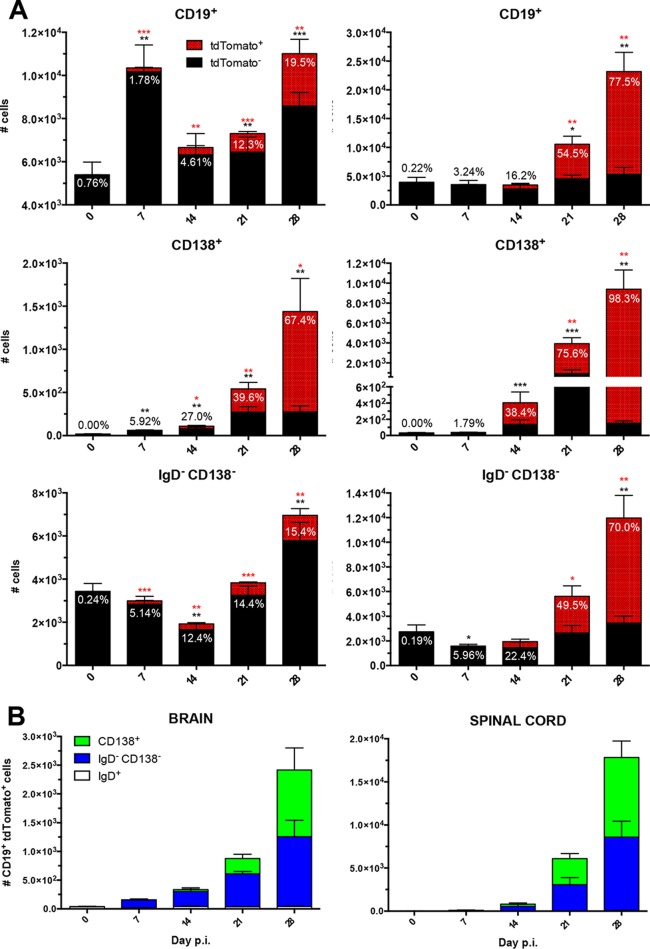
Virus-specific tdTomato^+^ ASC and Bmem accumulate progressively in the CNS subsequent to peripheral GC formation in MHV-infected mice. (A) Numbers of total CD19^+^ B cells, CD138^+^ ASC, IgD^−^ CD138^−^ Bmem, and IgD^+^ B cells in brains (left) and spinal cords (right). Data for tdTomato^−^ cells are represented in black, and data for tdTomato^+^ cells are shown in red; the relative percentage of tdTomato^+^ cells within each defined B cell population is indicated above or within the bar. (B) Numbers of CD19^+^ tdTomato^+^ cells are subdivided into CD138^+^ ASC, IgD^−^ CD138^−^ Bmem, and IgD^+^ B cell subsets for brain and spinal cord. Data represent the mean values ± SEM for individual mice from 2 separate experiments, each comprising 3 to 4 individual mice per time point. Statistically significant differences from the results for naive mice (day 0 p.i.), determined by unpaired *t* test, are denoted as follows, with asterisks in red or black denoting significant differences in the tdTomato^+^ or total population, respectively: *, *P* < 0.05; **, *P* < 0.01; ***, *P* < 0.001.

**FIG 6:**
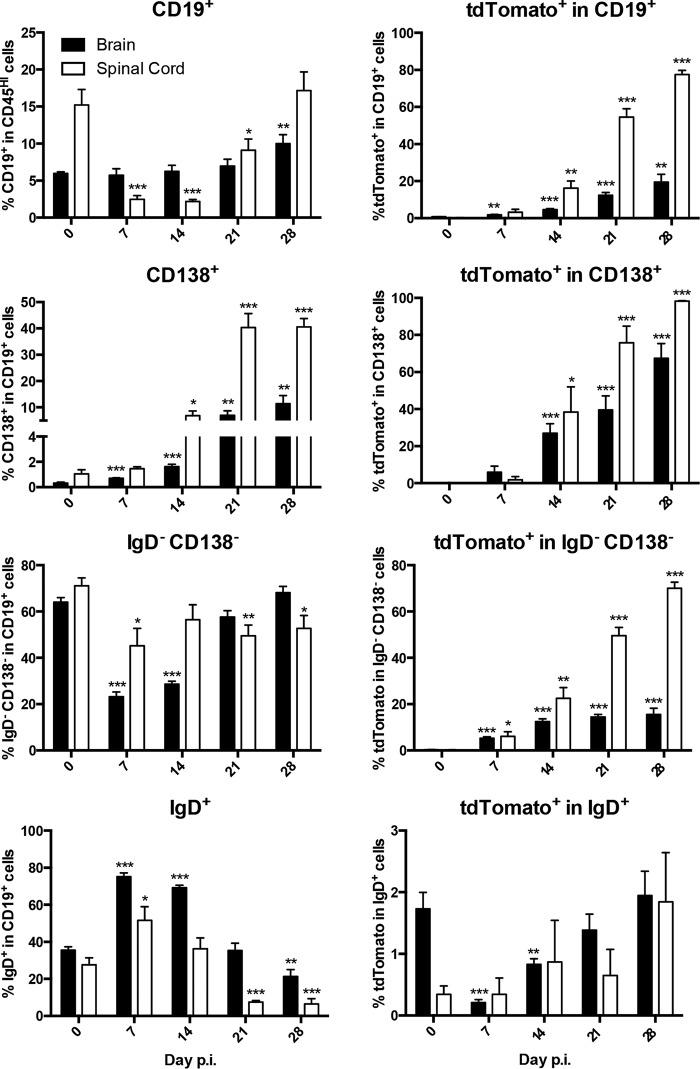
Virus-specific tdTomato^+^ B cells progressively dominate the ASC and Bmem subsets in spinal cords throughout viral persistence. Left, frequencies of CD19^+^ B cells within total brain or spinal cord CD45^HI^ infiltrating cells and the relative proportions of indicated B cell subsets within the CD19^+^ B cell population. Right, frequencies of tdTomato^+^ cells within the indicated B cell populations in brain or spinal cord. Data represent the mean values ± SEM for individual mice from 2 separate experiments, each comprising 3 to 4 individual mice per time point. Statistically significant differences from the results for naive mice (day 0 p.i.), determined by unpaired *t* test, are denoted as follows: *, *P* < 0.05; **, *P* < 0.01; ***, *P* < 0.001.

To assess how the kinetics of tdTomato^+^ B cells in CLN correlate with their accumulation within the CNS, both brains and spinal cords were analyzed for B cell phenotypes by flow cytometric analysis. Both organs harbored low numbers of CD19^+^ B cells in naive mice, but the frequency of tdTomato^+^ B cells was negligible. While brains only showed significant increases in total B cells at days 7 and 28 p.i., spinal cords started showing an increase at day 21 p.i., which progressed to 5-fold-higher numbers compared to the numbers in naive controls. Furthermore, tdTomato^+^ cells emerged in both brains and spinal cords at day 14 p.i. and progressively increased throughout day 28 p.i. (Fig. 5 and 6). However, whereas the proportion of tdTomato^+^ cells within the total CD19^+^ population did not exceed 20% in brains, it reached nearly 80% in spinal cords at day 28 p.i. (Fig. 5 and 6). CD138^+^ ASC started to accumulate at day 14 p.i. and increased progressively out to day 28 p.i. in both brains and spinal cords (Fig. 5 and 6). Furthermore, the percentage of tdTomato^+^ cells within ASC increased more extensively within spinal cords than in brains, reaching ∼98% compared to ∼70%, respectively, by day 28 p.i. (Fig. 5A and 6).

IgD^−^ CD138^−^ Bmem numbers in brains and spinal cords remained unaltered or even decreased compared to the numbers in naive mice during acute infection out to day 14 p.i. Brains only showed a marginal increase in Bmem at day 21 and more significant elevation by day 28 p.i. However, the relative proportion of tdTomato^+^ cells remained <20% (Fig. 5A). In contrast, IgD^−^ CD138^−^ Bmem in spinal cords were significantly elevated at day 21 p.i. and reached 6-fold-higher levels than in naive mice by day 28 p.i. Distinct from the results for brains, the percentage of tdTomato^+^ cells was already higher at day 14 p.i. and increased progressively, reaching ∼70% by day 28 p.i. (Fig. 5A and 6). The differences in the proportions of tdTomato^+^ B cells were thus much more pronounced in Bmem than in ASC when comparing brain and spinal cord populations. These data suggested that viral-Ag-experienced, GC-derived B cells accumulated preferentially in spinal cords, potentially outcompeting bystander or less differentiated B cells. Similar to the periphery, the frequency of tdTomato^+^ cells within IgD^+^ B cells remained <2% and never exceeded 1 × 10^2^ cells in either CNS tissue (Fig. 5B and 6).

Comparison of total numbers of tdTomato^+^ cells and their compositions of ASC and Bmem in brains and spinal cords over time following infection revealed overall similar kinetics of virus-specific B cell accumulation (Fig. 5B). However, as indicated above, the spinal cord harbored vastly more tdTomato^+^ B cells at 21 and 28 days p.i. Moreover, the vast majority of tdTomato^+^ B cells in the CNS were IgD^−^ CD138^−^ Bmem out to day 21 p.i., reflecting their dominance in CLN. However, irrespective of their low proportions in CLN throughout persistence, ASC increased in both brains and spinal cords, resulting in proportions approximately equal to those of Bmem by day 28 p.i. (Fig. 5B).

### Virus-specific tdTomato^+^ B cells localized to the CNS parenchyma.

**FIG 7:**
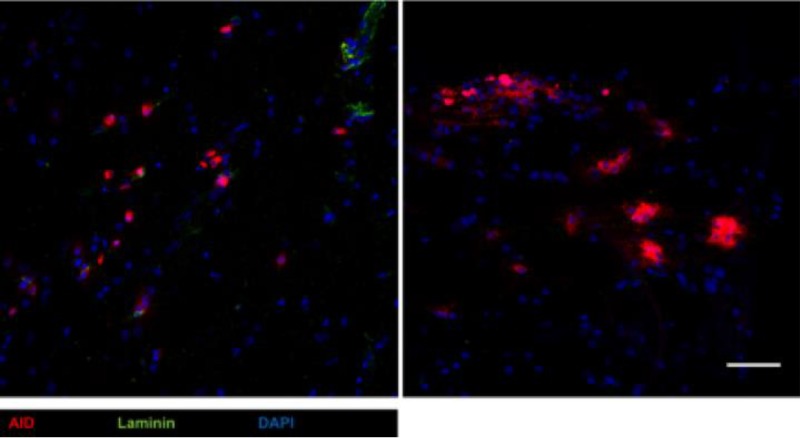
Virus-specific tdTomato^+^ cells are concentrated in focal white matter tracks along the spinal cord, but rarely form clusters. Representative fluorescent immunohistochemical images of longitudinal spinal cord sections at day 28 p.i. stained for basement membrane laminin (green), nuclear stain DAPI (blue), and endogenous tdTomato expression (red), taken at ×40 magnification from projected Z-stacks of 6.69 μm in depth. The left image illustrates the representative scattered distribution of tdTomato^+^ cells, while the right image depicts a rare cluster of tdTomato^+^ cells. Images are representative of the results for 2 mice. Scale bar = 50 μm.

Previous histological examination of B cell localization within the CNS following infection with a nonlethal variant of MHV-JHM revealed CD138^+^ cells, as well as IgM^+^ and IgG^+^ cells, scattered prominently within white matter or perivascular sites (23, 29). Moreover, in the brain, more differentiated IgG^+^ B cells preferentially accumulated in the parenchyma with a scattered rather than clustered pattern (1), while IgD^+^ cells appeared restricted to meningeal and perivascular sites. Histological evaluation of spinal cords for tdTomato^+^ cells at day 21 p.i. supported prominent localization to white matter tracks (Fig. 7). Moreover, tdTomato^+^ cells were not associated with laminin-positive areas marking perivascular matrix and were found in select areas of the spinal cord, where they exhibited a scattered distribution; tdTomato^+^ cell clusters were very rare.

### Virus-specific B cells were continually recruited to the infected CNS.

**FIG 8:**
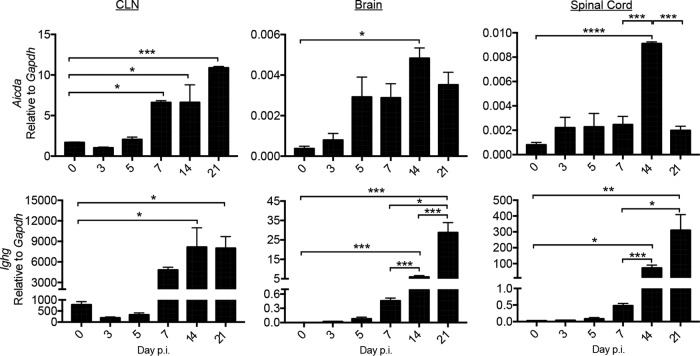
Transcript levels of B cell differentiation-associated genes in the CLN and CNS over the course of MHV-A59 infection. CLN, brain, and spinal cord tissue were assessed for *Aicda* (AID, top) and *Ighg* (IgG, bottom) mRNA levels by real-time PCR over time. Data are expressed as the mean transcript level ± SEM relative to the results for *Gapdh* mRNA for ≥3 mice per time point per group from 2 separate experiments. Statistically significant differences between the results for wild-type and CD19^−/−^ mice are denoted as follows: *, *P* < 0.05; ***, *P* < 0.001; ***, *P* < 0.001; ****, *P* < 0.0001.

The data described above demonstrated that virus-specific tdTomato^+^ B cells progressively accumulated in the CNS coincident with mature GC formation at days 14 to 28 p.i. However, it remained unclear whether they were continuously recruited from ongoing peripheral GC reactions or underwent further affinity maturation and differentiation within the CNS during persistent infection. Neither ASC nor Bmem isolated from the MHV-JHMv2.2-1-infected CNS at day 21 p.i. exhibited detectable *Aicda* transcripts, indicating that these cells were not undergoing SHM within the CNS (2). To confirm sparse if any *Aicda* mRNA expression in the CNS of tamoxifen-treated, MHV-A59-infected AID^Cre^-Rosa26^tdTomato^ mice, in which AID is only expressed from one allele (4), we monitored *Aicda* mRNA levels in CLN relative to the levels in brains and spinal cords throughout infection (Fig. 8). *Ighg* mRNA levels were assessed as a measure of ASC IgG secretion (2). In CLN, *Aicda* mRNA levels increased between days 5 and 7 p.i. and were further elevated by day 21 p.i., corresponding with GC formation. In contrast, although a statistically significant increase in *Aicda* mRNA was evident in brains and spinal cords at day 14 p.i., the levels were overall ∼4 orders of magnitude (10^4^) lower than in CLN. *Ighg* mRNA levels increased prominently in CLN by day 14 p.i., reflecting GC-matured ASC. In the CNS, *Ighg* mRNA levels also did not increase significantly until day 14 p.i., and the levels increased further by day 21 p.i. Moreover, the levels were ∼10-fold higher in spinal cords than in brains, consistent with higher IgG secretion. These mRNA kinetics were similar to those in MHV-JHMv2.2-1-infected mice (2). While *Ighg* mRNA levels were overall lower in the CNS than in CLN, the ∼100-fold-higher ratio of *Aicda* relative to *Ighg* mRNA levels in CLN versus spinal cords (∼1 × 10^−3^ versus 5 × 10^−5^, respectively) confirmed minimal local AID activity in the CNS.

**FIG 9:**
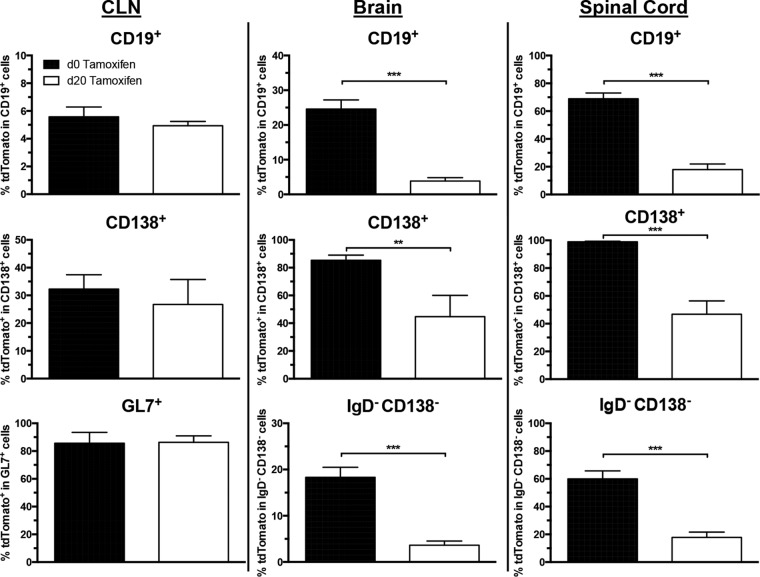
Virus-specific tdTomato^+^ B cells are continually recruited to the persistently infected CNS. Infected AID^cre^-Rosa26^tdTomato^ mice were treated with tamoxifen starting either at day 0 (black bars) or day 20 p.i. (open bars), and frequencies of tdTomato^+^ cells within the indicated B cell populations from CLN, brains, and spinal cords determined at day 28 p.i. Data represent the mean values ± SEM for individual mice from 2 separate experiments, each comprising 2 to 3 individual mice per time point and group. Statistically significant differences between the results for experimental groups, determined by unpaired *t* test, are denoted as follows: *, *P* < 0.05; **, *P* < 0.01; ***, *P* < 0.001.

To support ongoing recruitment from protracted GC reactions typical of viral infections, we initiated administration of tamoxifen to infected AID^Cre^-Rosa26^tdTomato^ mice at day 20 p.i. This approach ensures that only those B cells primed or undergoing continuous differentiation after day 20 p.i. are marked by tdTomato expression. Any accumulation of tdTomato^+^ B cells in the CNS after day 20 p.i. would imply that these cells were derived from protracted peripheral GC reactions sustained during the chronic phase. CLN, brain, and spinal cord cells were thus assessed for the presence of tdTomato^+^ ASC and Bmem cells by flow cytometry 8 days after initial tamoxifen administration (day 28 p.i.). Slightly reduced frequencies of tdTomato^+^ B cells within total CD19^+^ B cells in CLN in mice receiving tamoxifen starting at day 20 p.i. compared to those starting at day 0 p.i. did not reach statistical significance (Fig. 9). Furthermore, mice receiving tamoxifen treatment at day 20 p.i. revealed a frequency of ∼30% tdTomato^+^ cells in the CD138^+^ ASC population, similar to the frequency in mice receiving tamoxifen continuously from day 0 p.i. (Fig. 9), indicating that nearly all the virus-specific ASC present in the CLN during chronic disease had undergone affinity maturation after day 20 p.i. in established GCs. Importantly, the frequencies of tdTomato^+^ cells within the GL7^+^ GC phenotype B cells were similar, at ∼80% (Fig. 9). These results support continual selection and turnover of virus-specific B cells in the CLN, consistent with ongoing GC reactions during chronic infection.

In the brains and spinal cords of infected mice, the frequency of tdTomato^+^ cells in total CD19^+^ B cells was significantly decreased in mice receiving tamoxifen beginning at day 20 p.i. compared to the frequency in mice receiving treatment from day 0 p.i. (Fig. 9). The frequencies of CD19^+^ B cells within CD45^HI^ infiltrates were not significantly different between treatment groups in either the brains or spinal cords (data not shown). Nevertheless, these mice exhibited appreciable accumulations of tdTomato^+^ B cells, reaching 10% and 20% of the frequencies observed in the control group in the brains and spinal cords, respectively (Fig. 9). Interestingly, the reductions in tdTomato^+^ populations were more prominent in IgD^−^ CD138^−^ Bmem than in CD138^+^ ASC in both brains and spinal cords. Overall, these results are consistent with ongoing peripheral activation, differentiation, and migration of virus-specific ASC and Bmem to the CNS during chronic infection.

## DISCUSSION

Production of virus-specific Ab within the CNS is associated with protection during infection with RNA viruses (24, 34). Furthermore, although both IgM^+^ and IgG^+^ B cells are recruited to the CNS, their specificity or derivation from ongoing GC reactions remains largely unexplored. We therefore infected AID^Cre^-Rosa26^tdTomato^ reporter mice with MHV-A59 to monitor the expansion and distribution of B cells having undergone SHM as an indicator of viral-Ag-induced activation. Our results demonstrate that tdTomato^+^ B cells in draining lymph nodes were already evident at day 7 p.i., prior to peak GL7^+^ activated and GC B cells, as well as defined GC structures evident at day 14 p.i. This early, GC-independent, tdTomato^+^ population was comprised of similar proportions of CD138^+^ ASC and IgD^−^ CD138^−^ Bmem. Moreover, CD138^+^ tdTomato^+^ ASC peaked at day 7 p.i., accounting for ∼65% of total ASC. While the early expansion of ASC prior to GC formation confirmed results in Blimp-GFP mice infected with the more pathogenic, sublethal MHV-JHMv2.2-1 variant (23), these ASC were previously interpreted to result from innate bystander activation, as they were undetectable in virus-specific ELISPOT assays (14). However, their high frequency of tdTomato expression indicates that these ASC are viral Ag specific but produce Ab with insufficient affinity to be detected by virus-specific ELISPOT. This notion is supported by MHV-JHMv2.2-1 infection of CD19-deficient mice (14), in which a significant reduction in early ASC within CLN at day 7 p.i. was consistent with the function of CD19 to lower the Ag-driven B cell activation threshold. Interestingly, somatic mutations introduced by AID may not be viral Ag selected but may still be specific, as previously reported in a subset of long-lived, GC-independent, IgM-secreting ASC induced by vaccination or peripheral infection (12). Nevertheless, independent of SHM evident at day 7 p.i., these ASC do not appear to be migration competent, as tdTomato^+^ ASC do not emerge in the CNS until day 14 p.i. Irrespective of migration capacity, these results are consistent with preferential retention of Ag-induced, long-lived IgM ASC in the spleen following peripheral immunization or infection (12).

While small numbers of tdTomato^+^ B cells emerged in the CNS during acute infection, their CNS accumulation accelerated substantially out to day 28 p.i. following the formation of peripheral GCs at day 14 p.i. Mature GCs in CLN were sustained out to at least day 28 p.i. (data not shown), but tdTomato^+^ B cell numbers declined after day 14 p.i., consistent with the death of low-affinity clones during ongoing affinity maturation and/or egress from lymphoid tissue into circulation. The progressive increase in tdTomato^+^ B cells in the CNS thus supported the idea of ongoing egress of GC-matured B cells from CLN and subsequent migration to the persistently infected CNS. Overall, these results suggested that GCs are important for licensing migration of Ag-specific B cells to sites of inflammation. The idea of imprinting of migratory capacity by GCs was supported by the increasing percentages of tdTomato^+^ cells within the ASC and Bmem populations, which ultimately represented a majority within both subsets. However, a lower proportion of tdTomato^+^ cells within Bmem in brains compared to the proportion in spinal cords suggests that additional events dictated by the regional CNS environment control the recruitment or survival of Ag-specific B cells. Overall, the preferential accumulation of tdTomato^+^ B cells throughout persistence demonstrated that recently activated B cells display enhanced CNS migratory capacity compared to that of preexisting, non-MHV-specific ASC or Bmem with heterologous specificity.

The relative distributions of Ag-specific ASC and Bmem within the priming and effector sites have not been characterized during any neurotropic infection to our knowledge. In CLN, ASC and Bmem within the tdTomato^+^ B cell population were distributed similarly prior to GC formation. Moreover, the vast majority of GL7^+^ cells were tdTomato^+^ at day 7 p.i, indicative of an activated pre-GC phenotype. Similar to GC-independent, IgM-secreting ASC with Ag-induced SHM (12), GC-independent, unswitched as well as switched Bmem have also been described during the early primary response to R-phycoerythrin (R-PE) immunization (33). However, GL7^+^ cells were sparse at day 7 p.i., and their increase by day 14 p.i. coincided with overt GC formation. At this time, the composition of tdTomato^+^ B cells was prominently skewed toward Bmem, with the proportion of ASC dropping below 10% after day 14 p.i., despite a more mature GC structure.

The relative abundances of virus-specific tdTomato^+^ Bmem and ASC in both brains and spinal cords largely reflected those in draining LN at days 14 and 21 p.i., with tdTomato^+^ Bmem prevailing over ASC. Surprisingly, however, ASC accounted for nearly 50% of tdTomato^+^ B cells by day 28 p.i. The steadily increasing proportion of ASC in the CNS by day 28 p.i. suggested more efficient CLN egress and/or migration of ASC than of Bmem during ongoing GC reactions. These findings are overall consistent with a temporal switch observed in the output of GCs following model Ag immunization: GCs are initially dedicated to generating Bmem and only switch to ASC output after peak Bmem expansion (35). However, limiting viral Ag in CLN during persistence may reduce ASC over Bmem output in CLN. Nevertheless, the progressive ASC emergence relative to that of Bmem in the CNS over time supports the notion that early GC reactions are dedicated to preferential production of Bmem with the capacity to migrate to the CNS.

ASC are known to have a CXCR4-dependent migratory window, when they preferentially home to the bone marrow following peripheral immunization or infection (36–39). However, CXCR3/CXCL10 interactions are essential in mediating ASC migration and access to the CNS parenchyma (23). In contrast, Bmem remain prominent in secondary lymphoid organs and recirculate (40, 41). While Bmem accumulating in the CNS are characterized by the expression of chemokine receptor CCR7 (2), the chemokines essential for their CNS migratory behavior *in vivo* have not been characterized. Differential chemokine receptor responsiveness during protracted GC reactions may thus further imprint preferential CNS recruitment of ASC during ongoing viral persistence.

Ongoing egress of tdTomato^+^ B cells from CLN during late GC reactions was indeed supported by tamoxifen administration at day 20 p.i. Analysis of B cells 8 days later revealed that ∼20% of tdTomato^+^ B cells were derived from B cells initiating AID-driven tdTomato expression after day 20 p.i. Their derivation from the periphery was supported by similar percentages of tdTomato^+^ cells within total CD19^+^ B cells, GL7^+^ B cells, and CD138^+^ ASC at day 28 p.i., independent of the administration of tamoxifen throughout acute infection or starting at day 20 p.i. Whether these tdTomato^+^ B cells are derived from cells already expressing AID and undergoing further affinity maturation after day 20 p.i. or from newly primed B cells recruited to GCs remains to be determined. Nevertheless, the less prominent reductions of tdTomato^+^ cells in ASC compared to the reductions in Bmem in both brains and spinal cords following late compared to early tamoxifen treatment is consistent with the notion that ongoing recruitment primarily involves ASC still undergoing SHM after day 20 p.i. These data are also consistent with preferential output of migratory ASC by day 28 p.i. We have excluded *de novo* AID expression within the CNS based on previous analysis of mRNA transcript levels from IgD^−^ CD138^−^ Bmem and CD138^+^ ASC isolated from the CNS at 21 days p.i. (2), as well as IgD^+^ B cells at day 7 p.i. Nevertheless, we cannot exclude the possibility that the relative increase in ASC over Bmem in the CNS may also be driven by local conversion of Bmem into ASC by persistent viral Ag, ongoing pattern recognition receptor mediation, or cytokine stimulation.

Overall, our data demonstrate for the first time that virus-specific B cells exhibiting SHM only emerge in the CNS following peripheral GC formation. Moreover, the accumulation of ASC and Bmem appears to be regulated by temporal events within GCs, as well as CNS regional cues during viral persistence. While early GC reactions supported preferential Bmem accumulation in the CNS, late GC reactions preferentially mediated ASC migration to the CNS. Importantly, virus-specific B cells expressing AID in peripheral GCs sustained during CNS viral persistence are continually recruited to the chronically infected CNS. While these data do not support involvement of bystander recruitment of preexisting Bmem or ASC with irrelevant specificity to CNS inflammation, they do predict that sustained GC reactivity may recruit *de novo* B cell activation to Ags released from damaged tissue. Such B cells may thus give rise to autoreactive Abs or promote autoreactive T cells, as recently demonstrated in multiple sclerosis (42).

## MATERIALS AND METHODS

### Mice, virus infection, and tamoxifen administration.

C57BL/6 mice were purchased from Charles River Laboratories (Wilmington, MA). Aicda^CreERT2^ mice and Rosa26^loxp-tdTomato^ mice [B6.Cg-*Gt*(*ROSA*)*26Sor^tm14^*^(^*^CAG-tdTomato^*^)^*^Hze^*/J; Ai14] were generously provided by Claude-Agnes Reynaud and Dimitrios Davalos, respectively, and have been previously described (4). Progeny of the cross between AID^CreERT2^ and Rosa26^loxp-tdTomato^ mice were termed AID^Cre^-Rosa26^tdTomato^ mice. Mice were housed at the Cleveland Clinic Lerner Research Institute under pathogen-free conditions. All animal procedures were executed in accordance with approved guidelines by the Cleveland Clinic Lerner Research Institute Institutional Animal Care and Use Committee. Male and female mice of 6 to 8 weeks of age were infected by intracranial (i.c.) injection with 2,000 PFU of hepato- and neurotropic MHV-A59, kindly provided by Volker Thiel (University of Bern, Bern, Switzerland) (43). Infected animals were evaluated daily for clinical signs utilizing the following scale: 0, healthy; 1, hunched back and ruffled fur; 2, inability to correct to upright position or partial hind limb paralysis; 3, complete hind limb paralysis and wasting; 4, moribund or deceased. To induce tdTomato expression, mice were administered 3 mg of tamoxifen (Sigma-Aldrich, St. Louis, MO) dissolved in corn oil (20 mg/ml) via oral gavage every other day. Treatment was initiated at day 0 p.i. unless otherwise stated.

### Mononuclear cell isolation and flow cytometric analysis.

Cells were isolated from the CNS as described previously (13, 44). Briefly, brains or spinal cords harvested from phosphate-buffered saline (PBS)-perfused mice were mechanically homogenized in Dulbecco’s PBS using ice-cold Tenbroeck grinders. The resulting suspensions were centrifuged at 450 × *g* for 7 min at 4°C, supernatants stored at −80°C for subsequent analysis, and cells resuspended in RPMI medium. Cells were adjusted to 30% Percoll (Pharmacia, Piscataway, NJ), underlaid with 1 ml 70% Percoll, and collected from the 30%–70% Percoll interface following centrifugation at 850 × *g* for 30 min at 4°C. After washing, cells were resuspended in fluorescence-activated cell sorter (FACS) buffer (0.5% bovine serum albumin [BSA] in phosphate-buffered saline [PBS] containing a 10% serum mixture comprised of mouse, goat, and horse serum [1:1:1] and rat anti-mouse FcγIII/II monoclonal Ab [MAb] [2.4G2; BD Bioscience, San Diego, CA]) for 20 min on ice. Cells were then stained with MAb specific for cell surface markers CD45 (30-F11), CD19 (1D3), CD138 (281-2), T cell and B cell activation antigen (GL7) (all from BD Pharmingen), and IgD (11-26c; Affymetrix, Inc., San Diego, CA). Cells were then washed with FACS buffer, fixed with 2% paraformaldehyde, and analyzed on a BD LSR II or FACSAria III flow cytometer. The resulting data were analyzed with FlowJo software (Tree Star, Inc., Ashland, OR).

### ELISPOT.

ASC were determined by enzyme-linked immunosorbent spot assay (ELISPOT) as described previously (1, 13, 14). Briefly, sterile white 96-well filter plates with 0.45-μm-pore-size hydrophobic polyvinylidene difluoride (PVDF) membrane (Merck Millipore, Billerica, MA) were stripped with 70% ethanol for 2 min, washed with 0.1 M sodium bicarbonate buffer, and coated with undiluted MHV-A59 (∼9 × 10^6^ PFU/well) supernatant for approximately 16 h at 4°C. Wells were washed with washing buffer (0.05% Tween 20 in 1× PBS) and blocked with 5% fetal calf serum (FCS) in RPMI medium for 2 h at 37°C. Blocking buffer was replaced with cell suspensions at serial dilutions in triplicate in RPMI medium. Plates were incubated at 37°C for 4 h and then washed thoroughly to remove cells. After the addition of biotinylated rabbit anti-mouse IgG (0.5 μg/ml; Southern Biotech, Birmingham, AL) or biotinylated goat anti-mouse IgM (1.2 μl/ml; Jackson ImmunoResearch, West Grove, PA), plates were incubated for ∼16 h at 4°C, subsequently washed with washing buffer, and incubated with streptavidin-horseradish peroxidase for 1 h at 25°C. Following washes with washing buffer and 1× PBS, respectively, diaminobenzadine (DAB) solution was added to visualize spots. Once spots had developed sufficiently, the reaction was stopped by flushing wells with H_2_0 and the plates left to dry in the dark. The plates were scanned and spots quantified via ImmunoSpot (Cellular Technologies Ltd., Shaker Heights, OH). Threshold criteria for spot size were defined as between 0.0009 and 0.2257 mm^2^.

### Immunohistochemistry.

CLN and spinal cords from PBS-perfused mice were snap-frozen in Tissue-Tek O.C.T. (optimum cutting temperature) compound (Sakura Finetex, Torrance, CA) and sectioned at 10 μm (CLN) or 12 μm (spinal cords) using a Leica CM3050 S cryostat (Leica Microsystems, Exton, PA). Slide-mounted tissue sections were fixed with 4% electron microscopy (EM)-grade paraformaldehyde (Electron Microscopy Sciences, Hatfield, PA) for 20 min, washed 3 times with 0.1% Triton X-100 (Sigma-Aldrich, St. Louis, MO) in PBS, and blocked with 5% BSA and 10% goat serum for 1 h. CLN were stained with rat anti-mouse B220 MAb (BD Biosciences, San Jose, CA) and hamster anti-mouse CD3 polyclonal Ab (BioLegend, San Diego, CA) overnight at 4°C. Sections were then washed with PBS and incubated with secondary Ab using Alexa Fluor 647 goat anti-rat and Alexa Fluor 488 goat anti-hamster Abs (Life Technologies, Grand Island, NY) for 1 h at room temperature. Spinal cords were stained as described above using rabbit anti-mouse laminin polyclonal Ab (Cedarlane, Burlington, NC) overnight at 4°C and Alexa Fluor 488 goat anti-hamster Ab (Life Technologies, Grand Island, NY) for 1 h at room temperature. Sections were mounted with Vectashield hard-set mounting medium with DAPI (4′,6-diamidino-2-phenylindole) (Vector Laboratories, Burlingame, CA) and examined using a Leica TCS SP5 II confocal microscope (Leica Microsystems, Exton, PA). Images were analyzed using Image J software (NIH; http://rsbweb.nih.gov/ij) implementing the FIJI plugin set (http://pacific.mpi-cbg.de/wiki/index.php/Fiji).

### Quantitative real-time PCR gene expression analysis.

CLN, brains, and spinal cords harvested from individual mice were snap-frozen, treated with 1 ml TRIzol (Invitrogen, Grand Island, NY), and homogenized using a TissueLyser and stainless steel beads (Qiagen, Valencia, CA). RNA was extracted according to the manufacturer’s instructions. DNA contamination was eliminated via DNase I treatment for 30 min at 37°C (DNA-free kit; Ambion, Austin, TX). cDNA was synthesized from RNA using Moloney murine leukemia virus (MMLV) reverse transcriptase (Invitrogen) and a 1:1 mixture of oligo(dT) primers and random primers (Promega, Madison, WI). Quantitative real-time PCR was performed using Applied Biosystems gene expression assays with TaqMan universal master mix on a 7500 fast real-time PCR system (Applied Biosystems, Foster City, CA). mRNA levels of glyceraldehyde 3-phosphate dehydrogenase (*Gapdh*), activation-induced cytidine deaminase (*Aicda*), and immunoglobulin gamma heavy chain (*Ighg*) were determined using TaqMan primers (Applied Biosystems). Transcript levels were calculated relative to the levels of the GAPDH housekeeping gene using the following cycle threshold (*C_T_*) formula: 2^[^*^CT^*^(GAPDH) −^ *^CT^*^(target gene)]^ × 1,000.

### Statistical analysis.

All results are expressed as the mean values ± standard errors of the means (SEM). Data were plotted and statistical significance determined utilizing GraphPad Prism 6 software. Statistically significant differences were determined by Student’s *t* test for *P* values of <0.05, <0.01, and <0.001, unless otherwise specified in figure legends.
